# Lumbar herniation following extended autologous latissimus dorsi breast reconstruction

**DOI:** 10.1186/1471-2482-13-16

**Published:** 2013-05-30

**Authors:** Sheila Margaret Fraser, Hiba Fatayer, Rajgopal Achuthan

**Affiliations:** 1 Department of Breast & General Surgery, Leeds General Surgery, Great George Street, Leeds LS1 3EX, UK

## Abstract

**Background:**

Reconstructive breast surgery is now recognized to be an important part of the treatment for breast cancer. Surgical reconstruction options consist of implants, autologous tissue transfer or a combination of the two. The latissimus dorsi flap is a pedicled musculocutaneous flap and is an established method of autologous breast reconstruction.

Lumbar hernias are an unusual type of hernia, the majority occurring after surgery or trauma in this area. The reported incidence of a lumbar hernia subsequent to a latissimus dorsi reconstruction is very low.

**Case presentation:**

We present the unusual case of lumbar herniation after an extended autologous latissimus dorsi flap for breast reconstruction following a mastectomy. The lumbar hernia was confirmed on CT scanning and the patient underwent an open mesh repair of the hernia through the previous latissimus dorsi scar.

**Conclusion:**

Lumbar hernias are a rare complication that can occur following latissimus dorsi breast reconstruction. It should be considered in all patients presenting with persistent pain or swelling in the lumbar region.

## Background

Breast reconstruction is an important part of the surgical treatment of breast cancer, and is usually performed following a mastectomy. Surgical options for reconstruction include implants, autologous tissue transfer or a combination of both. Autologous tissue transfer is often the preferred option as it can give a more natural appearance to the breast compared to implants alone [1,2]. The latissimus dorsi (LD) flap is an established method of autologous breast reconstruction with relatively few contraindications and complications [3-5].

Lumbar hernias are an unusual type of herniation of the postero-lateral abdominal wall. The majority of this hernia type is acquired, rather than congenital and occurs after surgery or trauma. The diagnosis is usually suspected on clinical grounds and confirmed by CT scanning [6,7].

Lumbar herniation after an LD flap is an uncommon complication that patients are not routinely warned about during the counseling and consent for the procedure. There have been very few published reports over the past 20 years regarding the incidence, detection and management of this complication [8,9].

We present the rare case of lumbar herniation following a latissismus dorsi reconstruction for breast cancer and the consequent diagnosis and treatment.

## Case presentation

A 63-year old female with a past medical history of bronchiectasis was diagnosed with a grade 3 ductal breast cancer. She initially underwent breast conservation surgery in the form of a right wide local excision and sentinel lymph node biopsy. Due to the proximity of the carcinoma to the surgical resection margins she subsequently had a skin sparing mastectomy and extended autologous latissimus dorsi flap reconstruction.

On her first post-operative outpatient visit she had a large seroma in relation to the tumour site, which was drained in clinic. She was seen two weeks later and was found to have a large seroma over the back wound (LD site); this was aspirated and drained 650 mls serous fluid. On two consecutive outpatient visits at fortnightly intervals she had recurrent large seromas at the LD site. On each occasion these were aspirated and over a litre of serous fluid drained.

**Figure 1:**
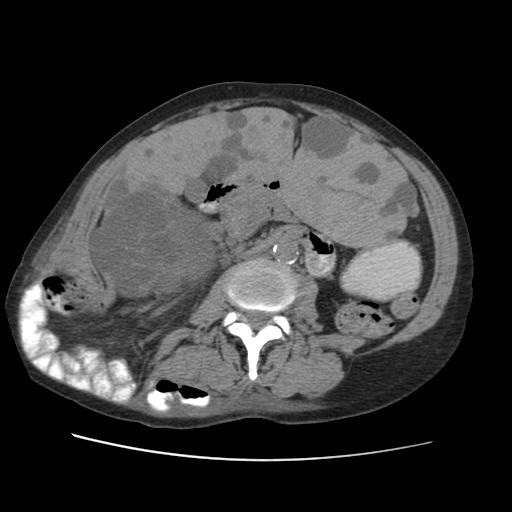
Axial CT scan demonstrating incisional lumbar hernia.

**Figure 2:**
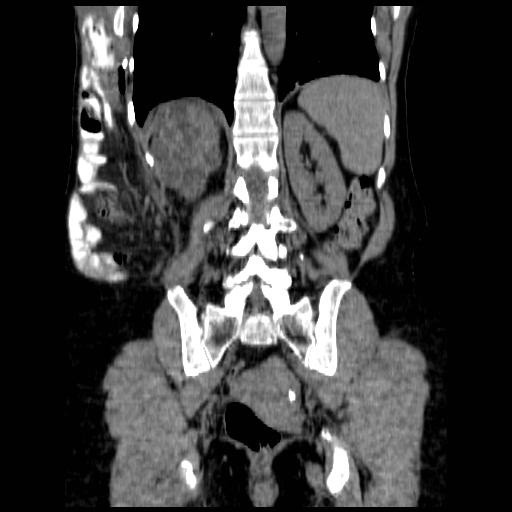
Coronal CT scan demonstrating incisional lumbar hernia.

On her fourth visit, following aspiration of the seroma there was a residual fullness in the lower region of the LD donor site. On palpation this felt like a separate firm lump, clinically in keeping with a possible lumbar hernia. An urgent CT scan of the abdomen and pelvis confirmed the diagnosis of a lumbar hernia containing the majority of the small bowel and right colon (Figures 1 and 2). The CT scan also demonstrated an incidental finding of an enlarged polycystic liver.

**Figure 3:**
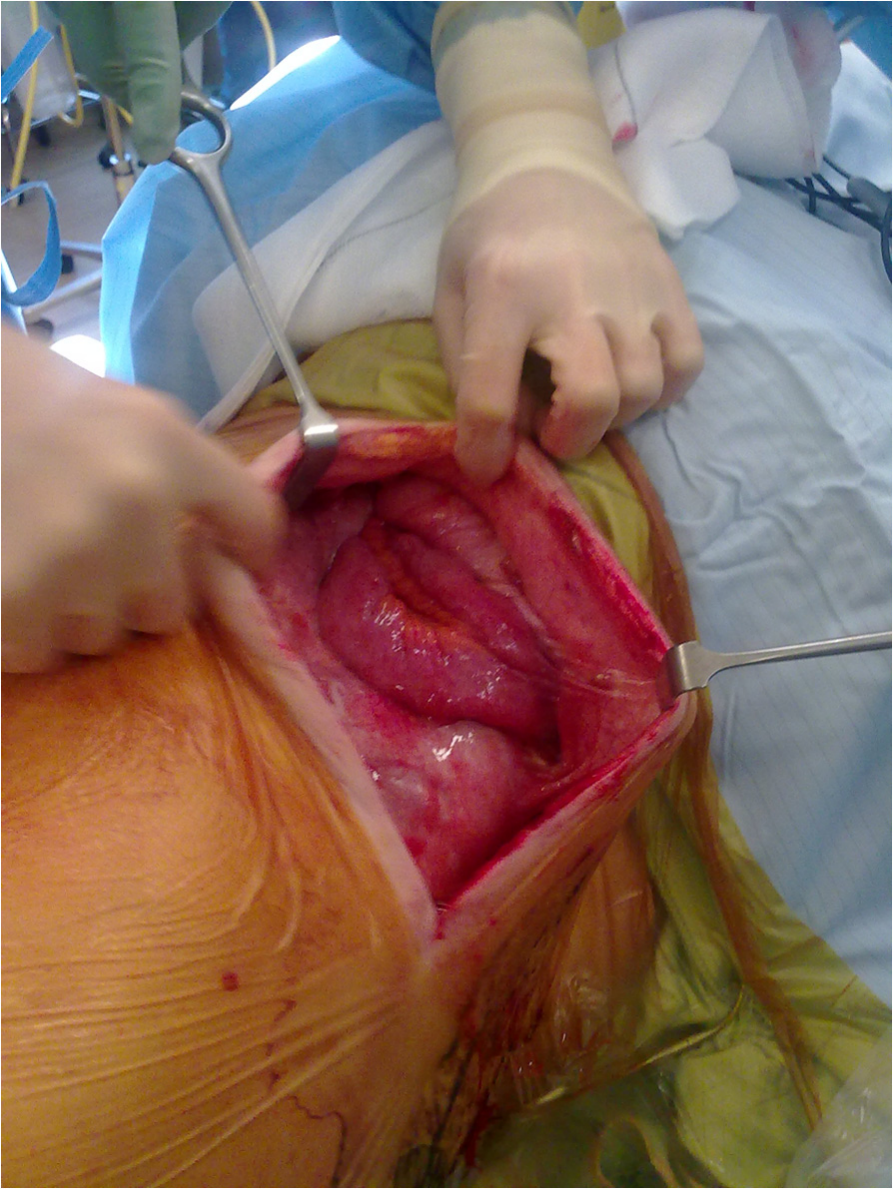
Intra-operative view of lumbar hernia, through previous latissimus dorsi scar.

She accordingly underwent an open repair of the lumbar hernia using the previous LD scar for access (Figure 3). Small bowel loops within the hernia sac were freed from adhesions and the small bowel and caecum reduced into the intra-abdominal cavity. The edges of the defect were closed with interrupted PDS, and a 30 × 30 cm onlay prolene mesh was used for reinforcement.

Postoperative recovery was uneventful and she was commenced on adjuvant hormonal and herceptin therapy.

## Discussion

Breast conservation surgery is an established method of treatment for early breast cancer. In the majority of patients this method of surgery, in combination with radiotherapy, provides effective oncological therapy and long-term survival as compared to mastectomy [10]. However over 30% women in the UK still require a mastectomy for the treatment of breast cancer. Reasons for mastectomy include large cancers, those directly behind the nipple, multi-focal cancers, patient preference and increasingly prophylactic mastectomies due to an improved understanding of the genetics of breast cancer [11,12].

Mastectomy is known to be associated with significant psychological consequences including a distorted body image, loss of self-esteem and sexual dysfunction. Breast reconstruction should be offered to virtually all women undergoing a mastectomy and is an integral part of the surgical treatment of breast cancer. The benefits of an immediate or delayed breast reconstruction are well recognized, for both emotional and physical well being [4]. Furthermore reconstruction techniques have been shown to be safe with minimal morbidity and no affect on local recurrence rates [5,13].

The aims of reconstructive surgery are to correct the anatomical defect after mastectomy and restore the shape and symmetry of the breasts. Surgical reconstruction options consist of implants, autologous tissue transfer or a combination of the two. In comparison to implants, autologous tissue transfer is considered to give a better aesthetic look [1,2,14].

The latissimus dorsi flap is a pedicled musculocutaneous flap, first used in the late 19th century to cover chest wall deformities following mastectomy complications. The current use of the latissimus dorsi flap was developed in the 1970s for breast reconstruction following mastectomy in patients with radiation damage to the skin and chest wall [15-17]. There are relatively few absolute contraindications to latissimus dorsi breast reconstruction. Complications can be split into flap complications and donor site complications, the most common being mastectomy skin flap necrosis and donor site seroma [18].

Lumbar hernias are an uncommon type of hernia, only 20% present as a congenital condition, the majority are acquired following surgery, trauma or inflammation [7]. Lumbar hernias typically are wide necked and therefore less likely to be prone to strangulation or obstruction. Lumbar hernias usually occur in 2 weak sites in the posterolateral abdominal wall – the superior (Grynfeltt-Lesshalft) and the inferior (Petit) lumbar triangles. However in large incisional defects the hernia can affect the entire lumbar region [6,19].

Due to its rarity there is no standardized surgical technique for lumbar hernia repair. When there is clinical suspicion of a lumbar hernia CT is recommended to get exact information on the size and content of the hernia and to plan for surgical repair [19].

Our patient had pre-disposing factors that in hindsight contributed to the development of a lumbar hernia, despite preservation of the lumbar fascia during the operation. The co-morbidities of bronchiectasis and a polycystic liver both reduced the intra-abdominal compartment size and increased the intra-abdominal pressure. This caused herniation of abdominal contents through a weakened area of the abdominal wall. On further questioning she admitted to a post-operative chest infection with associated coughing and had received oral antibiotics from her GP.

We currently do not routinely image seromas occurring in post-operative breast reconstruction patients. However there was clinical suspicion following recurrent aspiration, due to the residual fullness and the anatomical site of the seroma, that raised the suspicion of a lumbar hernia.

## Conclusion

Lumbar herniation is a rare complication that can occur following LD breast reconstruction. It should be clinically suspected in patients with a persistent swelling or pain in the lumbar region and subsequent CT scanning should be performed. Surgical repair is recommended in suitable patients; due to the large size these hernias can reach and related patient discomfort. Pre-existing co-morbidities should be carefully considered in all patients undergoing breast reconstruction.

### Consent

Written informed consent was obtained from the patient for publication of this Case report and any accompanying images. A copy of the written consent is available for review by the Series Editor of this journal.

## Abbreviations

CT: Computerized tomography; LD: Latissimus dorsi.

## Competing interests

The authors declare that they have no competing interests.

## Authors’ contributions

SMF wrote the background and discussion and revised the manuscript. HF wrote the case presentation. RA is the Consultant Surgeon who operated on the patient and gave final approval of the version to be published. All authors read and approved the final manuscript.

## Pre-publication history

The pre-publication history for this paper can be accessed here:

http://www.biomedcentral.com/1471-2482/13/16/prepub
